# Microwave-Assisted Pyrolysis of Carbon Fiber-Reinforced Polymers and Optimization Using the Box–Behnken Response Surface Methodology Tool

**DOI:** 10.3390/ma17133256

**Published:** 2024-07-02

**Authors:** Cynthie Dega, Rachid Boukhili, Babak Esmaeili, Jean-Philippe Laviolette, Jocelyn Doucet, Justine Decaens

**Affiliations:** 1 Department of Mechanical Engineering, Polytechnique Montréal, 2500 Chemin de Polytechnique, Montréal, QC H3T 1J4, Canada; 2 CTT Group, 3000 Avenue Boullé, Saint-Hyacinthe, QC J2S 1H9, Canada; 3 Pyrowave Inc., CP 174, Succ. Tour D/L Bourse, Montréal, QC H4Z 1C8, Canada

**Keywords:** carbon fiber-reinforced polymers (CFRPs), recycled carbon fiber-reinforced composites (rCFRCs), recycled carbon fiber-reinforced polymers (rCFRPs), recycling methods, microwave-assisted pyrolysis (MAP)

## Abstract

This article introduces an eco-friendly method for the reclamation of carbon fiber-reinforced polymers (CFRP). The research project involved numerous experiments using microwave-assisted pyrolysis (MAP) to explore a range of factors, such as the inert gas flow, the power level, the On/Off frequency of rotation, and the reaction duration. To design the experiments, the three-level Box–Behnken optimization tool was employed. To determine the individual and combined effects of the input parameters on the thermal decomposition of the resin, the data were analyzed using least-squares variance adjustment. The results demonstrate that the models developed in this study were successful in predicting the direct parameters of influence in the microwave-assisted decomposition of CFRPs. An optimal set of operating conditions was found to be the maximum nitrogen flow (2.9 L/min) and the maximum operating experimental power (914 W). In addition, it was observed that the reactor vessel’s On/Off rotation frequency and that increasing the reaction time beyond 6 min had no significant influence on the resin elimination percentage when compared to the two other parameters, i.e., power and carrier gas flow rate. Consequently, the above-mentioned conditions resulted in a maximum resin elimination percentage of 79.6%. Following successful MAP, various post-pyrolysis treatments were employed. These included mechanical abrasion using quartz sand, chemical dissolution, thermal oxidative treatment using a microwave (MW) applicator and thermal oxidative treatment in a conventional furnace. Among these post-treatment techniques, thermal oxidation and chemical dissolution were found to be the most efficient methods, eliminating 100% of the carbon black content on the surface of the recovered carbon fibers. Finally, SEM evaluations and XPS analysis were conducted to compare the surface morphology and elementary constitution of the recovered carbon fibers with virgin carbon fibers.

## 1. Introduction

Carbon fiber-reinforced polymer materials (CFRPs) have a diverse range of applications; for example, they have applications in the aerospace, aviation, wind turbine, automotive, construction, and sports industries [1,2,3,4]. The current demand for carbon fiber is approximately 142,500 tons per year on average [2], with North America accounting for 40% of this demand. Projections indicate that, by 2050, approximately half a million tons of CFRP waste will be generated globally, with the majority being produced in North America (162,000 tons) and Europe (145,000 tons) [3]. Numerous studies focus on the systematic disposal of carbon fiber composites at the end of their lifespan or during production, especially in the aerospace sector, where at least 30% of raw material is discarded and sent to landfills [4,5,6]. Despite this, carbon fibers represent high monetary value and have compelling mechanical properties, making them suitable for revalorization in various structural applications.

Since the early 1990s, various recycling technologies have emerged. These methods fall into three main categories: mechanical (grinding), thermal (cementitious incineration, pyrolysis, and gasification), and chemical (solvolysis) [7,8,9,10,11,12,13,14,15]. Among these techniques, pyrolysis stands out as the most efficient, as it requires less energy and yields recycled carbon fibers with properties comparable to virgin fibers [2]. Pyrolysis involves the decomposition of organic molecules in an inert or oxygen-poor atmosphere, typically at temperatures ranging from 400 to 1000 °C. It is generally an endothermic process that involves both exothermic and endothermic reactions. The endothermic phase is linked to primary cracking reactions, whereas the exothermic phase arises from secondary reactions between the vapor phase and the solid phase [16]. The pyrolysis process produces three types of products: syngas, oil, and solid residue [17]. The syngas is the gaseous fraction and can be used as an energy source for the process itself. The oil fraction can serve as fuel or chemical feedstock. The solid residue contains mostly intact carbon fibers that can be isolated and recovered for the production of new composite materials [17]. Principally, there are two types of pyrolysis, each based on a different heating mechanism: conventional heating through conduction or convection, and microwave-assisted pyrolysis (MAP). Both methods can convert CFRP waste into recycled carbon fibers (rCFs), pyrolytic oil, char, and non-condensable gases. Slow pyrolysis tends to produce more char, while fast pyrolysis yields less char and more pyrolytic oil [18]. Various factors, including the raw material, heating rate, temperature, and production process, influence the outcome of pyrolysis products [16,19,20,21]. Consequently, adjusting the pyrolysis parameters allows for the optimization of the resulting products according to their application.

Conventional heating, which involves heating the material from the outside to the inside, offers several advantages. Firstly, it causes the material to undergo progressive thermal cracking, promoting the production of char. This method facilitates the management of heterogeneous waste streams, including the production of materials with specific finishes and primers, without the need for chemical elements other than inert gases such as nitrogen and argon or certain catalysts. Furthermore, this process yields value-added products, including monomers or pyrolytic oils [15,22]. However, there are some drawbacks to consider. Pyrolysis requires substantial energy due to the high temperatures needed for thermal cracking, i.e., typically between 400 °C and 800 °C for CFRPs [23]. Moreover, it generates more volatile components compared to processes such as hydrolysis or methanolysis, potentially necessitating specialized equipment. Additionally, a liquid, such as water, is necessary for gas cooling or during gasification in the form of steam [18,22].

In microwave-assisted pyrolysis, the heating mechanism is different from conventional heating. Microwave heating functions by converting electromagnetic energy into thermal energy through the agitation of molecules exposed to microwaves. This conversion is facilitated by the polarization mechanism that occurs within the material [24]. There are four possible polarization mechanisms: electronic, atomic, dipolar, and interfacial [25]. During this process, electrons shift from their equilibrium position relative to the nucleus in an attempt to align with the electric field, which depends on the frequency being utilized. In typical domestic heating applications, a frequency of 2.45 GHz is employed, causing molecules to attempt to align with the electric field approximately 2.45 billion times per second (2.45 GHz) [25]. This alignment process generates friction between the molecules, which, in turn, is responsible for the heating effect. MAP not only combines most of the advantages associated with conventional pyrolysis but also offers additional benefits. Firstly, it allows five to ten times more waste to be processed for a given material due to the shorter residence times. For instance, in the case of polymers reinforced with carbon fibers, MAP takes only 7 min as compared to 2 to 6 h with conventional heating [26]. Another advantage is volumetric heating, wherein the material is heated from the inside rather than the outside as in conduction or convection heating [27]. This approach limits energy losses in comparison to conduction/convection heating, typically by around 20 to 30%. However, recycling multi-material composites using MAP is challenging, as these materials possess varying levels of permittivity. For example, conductive parts tend to reflect microwaves, resulting in the significant loss of electromagnetic energy during the reaction.

The pyrolysis of CFRP under an inert atmosphere is a temperature-dependent process [16,17,28]. In a microwave applicator that is controlled in power, the resultant temperature is the consequence of the selected level of power. Apart from parameters such as the level of power, temperature, and the feedstock being analyzed that could influence the yield of the pyrolysis reaction, the main advantage and high productivity of the microwave-assisted pyrolysis (MAP) process is due to the presence of micro-plasmas generated during microwave heating [20,21,29]. These micro-plasmas are extremely small, short-lived hotspots where temperatures are much higher than the average temperature of the surrounding material, causing the ionization of nearby molecules and facilitating pyrolysis [29]. An open question from a previous study [26] is the effect of these hotspots on the surface properties of the recycled carbon fibers. Actually, that first study was done in a fixed bed and the monitoring of the carbon fiber temperature during MAP revealed very high pics of temperature at certain locations. The first hypothesis for the current study would be that the introduction of the rotative bed would evenly distribute these hot spots across the matter and therefore provide a better temperature distribution (homogenized) during pyrolysis, in comparison to a fixed bed. Furthermore, the flow rate of the inert gas also has a role to play in that synergy. It is hypothesized that increasing the flow rate might also increase the extraction rate of devolatilized material, providing a better efficiency in comparison to a lower flow rate. However, too high of a flow rate might result in higher recycling costs. The aim of adjusting the power, the flow rate of the carrier gas, and the introduction of the rotative bed is to find an optimal design that promotes the occurrence of micro-plasma hotspots while ensuring even temperature distribution across the matter and a good extraction of the devolatilized material during pyrolysis.

In the screening phase, a set of parameters were initially considered. After preliminary experiments, four parameters that showed more influence on the pyrolysis reaction than the others will be analyzed in detail in this study. These four parameters are the nitrogen flow, the power level, the reaction time, and the On/Off rotation frequency. The On/Off rotation frequency is used to simulate either a full rotative bed (zero stop or no stop) or partial rotative bed (one, two, or four stops). Following CFRP MAP, four post-pyrolysis treatments were applied to remove the residual carbon black and non-reacted resin from the recovered carbon fibers surface, namely, thermal oxidation using a microwave (MW) applicator, thermal oxidation in a conventional furnace, mechanical abrasion using quartz sand, and chemical digestion. Thereafter, the pros and cons of each technique were investigated.

## 2. Materials and Methods

The CFRP material used for the pyrolysis experiments was expired prepreg roll TDS CYCOM 934 BMS 8-297 Plain weave provided by Composites M1, Laval, QC, Canada. The sulfuric acid (95–98%) and hydrogen peroxide (30%) used for the chemical digestion were CABDH30722.5LG and BDH7742-1, supplied by VWR Chemicals., Allentown, PA, USA. The aerospace virgin carbon fiber reference used in the XPS analysis was provided by the CTT group’s textile prototyping center, Saint Hyacinthe, QC, Canada and was scrap material from the braiding prototyping equipment (HTS40 Fiber). The equipment used for the pyrolysis process was provided by Pyrowave Inc., Montreal, QC, Canada. The pyrolysis and post-pyrolysis experiments and characterization methods, i.e., the TGA analyses and SEM observations, were conducted at the CTT Group laboratory, Saint Hyacinthe, Quebec, Canada. Finally, XPS analyses were conducted in the physical laboratory of the Ecole Polytechnique de Montreal, Montreal, Quebec, Canada. The characterization equipment and methods are described in Section 2.4. In addition, the equipment and consumables used for pyrolysis are listed below:

- Microwave oven (1100 W model);
- Spherical quartz flask (250 mL);
- Two thermocouples;
- Rotary evaporator;
- Data logger;
- Rotameter;
- Bath circulator;
- Ceramic tube.

**Figure 1:**
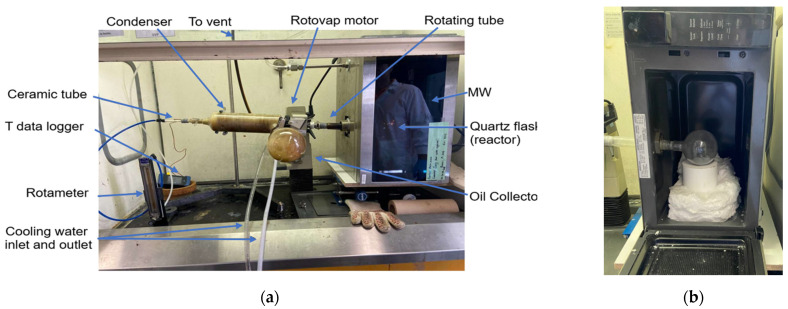
Experimental setup: (**a**) MW pyrolysis setup and (**b**) microwave cavity interior.

A Type-K thermocouple (ungrounded) was utilized to measure the temperature of the gas inside the quartz flask (the reactor), while a Type-J thermocouple was employed to monitor the temperature of the gas products at the outlet of the reactor. The flask was elevated using insulating glass wool and a Teflon bloc. These materials were selected for their dielectric properties. In fact, they exhibit excellent thermal insulating properties and they are nearly inert (transparent) to electromagnetic waves. A Buchi rotary evaporator RE111 (rotovap) from Buchi, DE, USA was used to rotate the flask and agitate the specimens to ensure a uniform distribution of microwaves in the reaction zone. The data logger monitored and registered the temperatures during the process. To purge the system, the ceramic tube injected nitrogen to remove oxygen from the flask, providing temperature homogeneity and removing the gaseous by-product of the pyrolysis reaction. The flow of nitrogen was controlled using a rotameter. The heavier gaseous by-products were liquified using the in-built diagonal Buchi rotovap condenser and collected in an oil collector flask. The light gases were vented through the fume hood. A bath circulator provided a constant flow of cold water to condense the by-products. Figure 1 shows the experimental setup used in this study.

### 2.1. Reclamation Procedure

The reclamation procedure began with sample preparation. The expired prepreg roll was unrolled and both the released paper and separation foil were cut and removed from the area to be sampled. Furthermore, the prepreg sheet was cut into small pieces, i.e., approximately 15 mm × 15 mm, and the quartz flask was weighed to the nearest 0.001 g. Then, approximately 15 g of cut specimen was loaded into the flask. Subsequently, the flask was securely placed in the microwave cavity via a connection to the rotameter tube through a chucking hole. The setup installation was completed by connecting two thermocouples, the carrier gas ceramic tube, the condenser, the data logger, the rotameter, and all of the corresponding adaptors. Once system installation was completed, the circulating bath was applied to provide cooling water and to condition the system. In addition, the rotary motor speed was adjusted to the desired value. Before applying the microwave and starting the pyrolysis reaction, the system was purged using a controlled flow of nitrogen for a minimum of 10 min. 

### 2.2. Design of Experiments via the Three-Level Box–Behnken RSM Tool

The Box–Behnken method is a design of experiments (DOEs) technique used to analyze and optimize complex systems with multiple variables. Its goal is to minimize the number of required trials while pinpointing the optimal input variable values for a given process [30]. Experimental runs were conducted based on a fractional factorial design generated using the Box–Behnken approach, with each variable set at three different levels, representing low, medium, and high values. This methodology allowed for the evaluation of the linear and quadratic effects of the factors on the target response [30]. In this study, the low, medium, and high values of the four influencing parameters (pyrolysis time, flow rate, power level, and On/Off rotation frequency) were determined during several preliminary experiments prior to microwave pyrolysis. A total of 17 experiments were conducted following a three-level Box–Behnken experimental design approach, with a designated range of variables as highlighted and classified in Table 1, Table 2 and Table 3. When the On/Off rotation frequency value X4 is not 0, the pause time between the rotation cycles is 30 s.

### 2.3. Post-Pyrolysis Treatments

After pyrolysis, a carbon black residue remains on the surface of the fibers. In addition, some resin remains unreacted if the pyrolysis reaction was not completed. Therefore, various methods were separately tested to remove this residual resin and carbon black with the aim of evaluating the advantages and disadvantages of each technique.

- Utilizing quartz sand abrasion in MAP

Quartz sand, which is also known as silica sand, is a naturally occurring mineral primarily composed of silicon dioxide (SiO_2_). It is a wear particle with dielectric properties that is suitable for the microwave applicator as it is mostly transparent to microwaves. The effect of sand particles is well-established in the literature. Moreover, they are commonly used in emerging technologies, especially for processes such as abrasion and machining [31,32,33,34,35,36,37]. In this study, Ottawa sand (CAS 14808-60-7) was used to simulate mechanical abrasion on the samples during microwave pyrolysis. The objective of this evaluation was to assess whether the use of quartz sand during CFRP pyrolysis would prevent the need for a chemical or thermal post-pyrolysis process. Two experiments were conducted using quartz sand during pyrolysis in the microwave applicator. The microwave pyrolysis protocol described in Section 2.1 was followed, with the only difference being that the quartz sand was weighed and introduced into the reactor along with the prepreg sample before pyrolysis. Accordingly, 50 g of quartz sand was placed in the reactor with 15 g of sample. In addition, the pyrolysis reaction was performed under the following conditions: 100% power, 914 W, 8 min, and 2.9 L/min of nitrogen flowrate. The Input power of the microwave applicator is 1100 W. The output power can be modulated using a control button labeled “DIAL POSITION”, which goes from 0 to 100%. Water calibration measurements were taken and made it possible to determine that the 100% position corresponds to 914 Watts on average. It should be noted that this is an experimental measurement, taken under laboratory conditions (21 °C, 50% RH) and that the temperature probe used can also introduce the phenomenon of reflectance, and therefore losses of energy by reflectance given that it is a metallic probe, which would have the effect of giving a result less than the true power received by the sample during the experiment.

b. Chemical dissolution using sulfuric acid and hydrogen peroxide mixture

The chemical digestion procedure described in ASTM D3171 procedure B was employed to remove the residual carbon black and non-reacted resin. In this procedure, sulfuric acid with a boiling point above 330 °C is used to digest the resin system over a short period of time. In this study, the effective mass fraction of fiber after pyrolysis was assessed as per ASTM D3171 B. This is one of the most accurate techniques to determine the mass fraction of carbon fiber in CFRP, as the hot sulfuric acid only digests the carbon black and the epoxy resin system and does not attack the carbon fiber. For each post-pyrolysis trial, the effective weight fraction of remaining resin (Wm) was determined and compared with the initial mass fraction of the resin in the prepreg.

c. Thermal oxidation

Two types of thermal oxidation, i.e., via the microwave applicator and using a conventional furnace, were performed under air after MAP. Conventional thermal oxidation was performed at 540 °C using a conventional furnace over 6 durations: 5, 10, 15, 20, 25, and 30 min. Microwave thermal oxidation was performed using the microwave applicator shown in Figure 1 at full power (914 W) at durations of 5, 10, and 15 min.

### 2.4. Characterization of Recycled Carbon Fiber (TGA, SEM, and XPS)

The weight fraction of the epoxy matrix and carbon fiber in the samples was estimated, both pre- and post-pyrolysis, using thermogravimetric analysis (TGA). TGA tests were conducted under a nitrogen atmosphere with a heating rate of 10 °C/min. This began at room temperature and went up to 1000 °C, followed by an isothermal hold for 1 h. The 1 h isothermal hold under nitrogen was followed by a return to 500 °C, after which heating under air was applied until 1000 °C for 30 min in order to simulate thermal degradation under air. After pyrolysis, the surface morphology of the recovered carbon fibers was examined using scanning electron microscopy (SEM). SEM imaging was performed using a HITACHI TM-1000 scanning electron microscope from HITACHI high-Tech, Toronto, ON, Canada. Furthermore, X-ray photoelectron spectroscopy (XPS) was performed on the recovered carbon fibers. The apparatus used for the XPS analysis was a VG ESCALAB 250Xi X-ray Photoelectron Spectrometer from Thermofisher, Waltham, MA, USA. A maximum depth of 10 nm was analyzed using XPS. 

## 3. Results and Discussion

### 3.1. Initial Characterization of CFRP Samples: TGA (Thermogravimetric Analysis) and Chemical Digestion

The digestion of the initial prepreg sample (per ASTM D3171 B) was performed to estimate the experimental weight fraction of carbon fiber (Wf) in the prepreg sample. Following CFRP sample digestion, the experimental average Wf value was determined to be 59.24 wt.%. Therefore, the deducted mass fraction of the resin, i.e., 40.76 wt.%, served as the baseline for the resin degradation comparison. 

**Figure 2:**
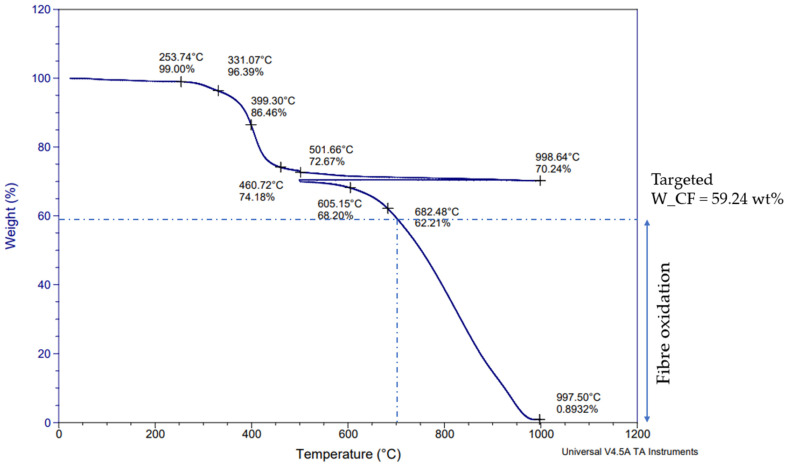
TGA of initial prepreg sample under nitrogen and then air.

**Figure 3:**
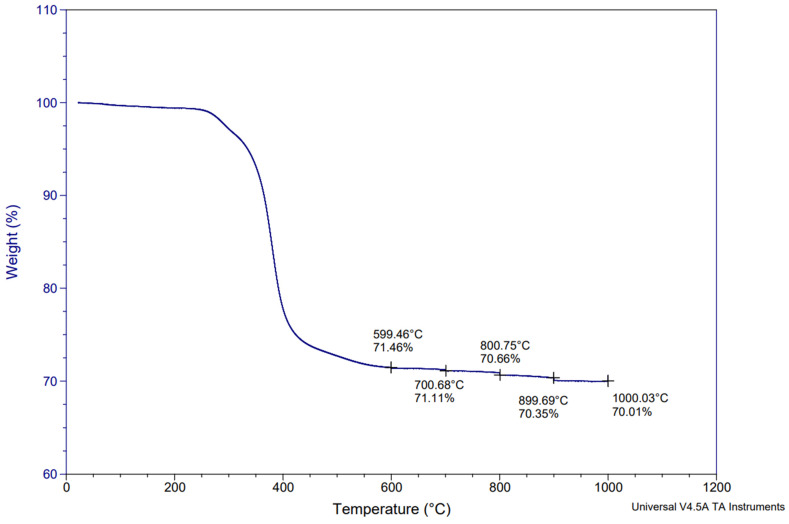
TGA of initial prepreg sample under nitrogen only.

The first TGA measurement was performed under nitrogen and then air, as shown in Figure 2. The second TGA was performed under nitrogen only, as shown in Figure 3. From Figure 3, the average pyrolytic carbon residue weight can be calculated as being 70.01%. This indicates that, in a nitrogen atmosphere, no further weight loss occurs during additional heating. In fact, CFRP pyrolysis under nitrogen results in pyrolytic carbon residues, which in this case are represented by the carbon fiber and the char on the surface of the carbon fibers. This pyrolytic carbon residue weight remains stable under nitrogen, even at high temperatures like 1000 °C, which correlates well with previous studies [28]. Furthermore, if this value is compared to the fiber content obtained by digestion (59.24%), the 10.76% difference can be attributed to char formation on the fiber surfaces due to the decomposition of epoxy resin during pyrolysis. Conversely, on Figure 2, this char is removed between 450 °C and 700 °C thanks to oxidation. Above 700 °C, it can be assumed that the fiber oxidation starts as the char is completely removed. Therefore, all of the post-pyrolysis oxidative treatments to recover clean carbon fibers shall be performed between 450 °C and 700 °C in order to avoid fiber oxidation. Figure 2 and Figure 3 illustrate the TGA curves obtained during the initial feedstock characterization. The noted points on Figure 2 and Figure 3 enable an assessment of the remaining weight during the thermal degradation of the samples, at selected temperatures. 

### 3.2. MAP of CFRP

Upon performing the 17 experiments as planned (Table 3), the results in Table 4 (expressed as percentages) were obtained, giving the following:

- Y1: the residual weight of CFRP;
- Y2: the weight lost from CFRP;
- Y3: the resin elimination percentage.

**Table 4:** Plan proposed by JMP software and corresponding results.

	Pyrolysis Time	N2 Flow Rate	MW Power	On/Off Frequency	Residual Weight of CFRP	Weight Lost from CFRP	Resin Elimination Percentage
Units	min	L/min	%	Number/run	%	%	%
Run	X1	X2	X3	X4	Y1	Y2	Y3
1	10	0.5	50	4	72.62	27.38	67.17
2	10	2.9	50	0	70.34	29.66	72.78
3	10	2.9	100	0	68.30	31.70	77.78
4	10	0.5	80	0	71.43	28.57	70.09
5	6	2.9	80	0	67.55	32.45	79.62
6	6	0.5	50	0	71.93	28.07	68.87
7	6	2.9	50	4	72.00	28.00	68.69
8	6	0.5	80	4	71.11	28.89	70.89
9	8	1.5	80	2	69.09	30.91	75.82
10	8	1.5	80	2	71.25	28.75	70.53
11	6	2.9	100	4	68.15	31.85	78.15
12	10	2.9	80	4	69.62	30.38	74.54
13	8	1.5	80	2	70.60	29.40	72.12
14	10	0.5	100	4	69.41	30.59	75.04
15	6	2.9	80	1	67.58	32.42	79.54
16	8	1.5	80	2	70.18	29.82	73.16
17	10	0.5	50	4	69.46	30.54	74.93

Example calculations for Y1, Y2, and Y3 are shown here for Run #1 (X1 = 10 min, X2 = 0.5 L/min, X3 = 50%, and X4 = 4):

$$Y1=\frac{W\_CFRP\_after\;pyrolysis}{W\_CFRP\_before\;pyrolysis}\;\times \;100$$

$$Y1\;(\mathrm{Run}\#1)=\frac{10.971\;g}{15.107\;g}\;\times \;100\;=\;72.62\%$$

Y2 (Run#1) = 1 − Y1 = 27.38%

$$Y3\;(\mathrm{Run}\#1)=\frac{Y2}{Wm}\;\times \;100$$

where W_m_ is the initial fraction of resin in the CFRP measured by chemical digestion, as per ASTM D3171 B. W_m_ was found to be 40.76%, as discussed in Section 3.1.

Therefore,

$$Y3\;(\mathrm{Run}\#1)=\frac{27.38}{40.71}\times 100$$

Y3 = 67.17%.

**Figure 4:**
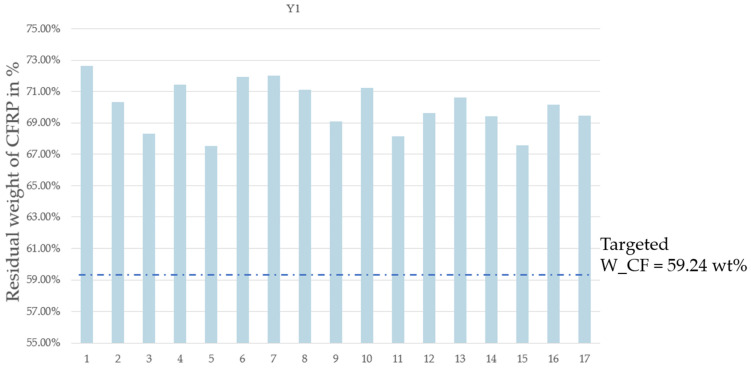
Residual weight of CFRP samples after MAP (Y1).

**Figure 5:**
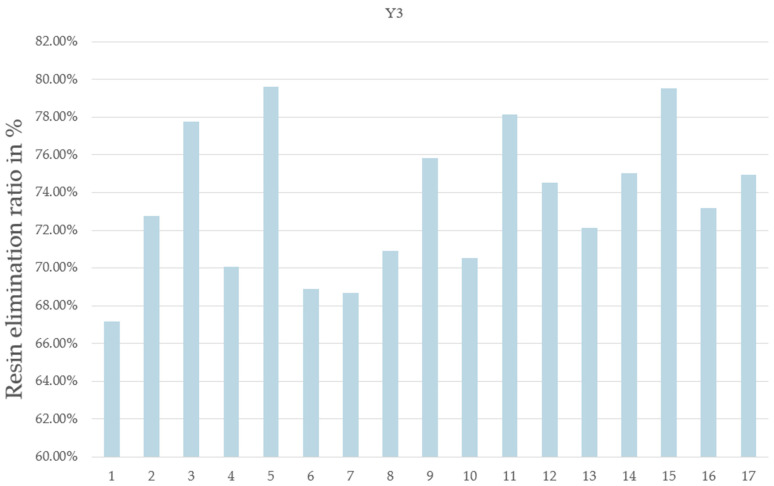
Resin elimination percentage (Y3).

The residual weight of the CFRP after pyrolysis, denoted as Y1, was estimated using the response surface methodology (RSM), which was designed using the Box–Behnken optimization tool. Parameter optimization for the four variables was carried out on the response values of resin elimination (Y3). The impact on resin elimination was analyzed and minimized by calculating the t ratio (Table 5) using the JMP software. The JMP software illustrates the importance of different parameters and their interactions. A parameter’s effect is deemed significant when the *p*-value is less than the t-value (Table 5). This analysis facilitates the identification of significant variables and the interactions among them. From the results obtained and considering the selected levels of the variables in this work, it can be concluded that the pyrolysis time and the On/Off rotation frequency are not significant in comparison to the two other parameters studied, i.e., power and carrier gas flow rate. Given the optimum value of the nitrogen flow rate and microwave power, it was possible to establish the optimum pyrolysis conditions as follows: maximum power level (100% power = 914 W), maximum nitrogen flow 2.9 L/min, non-stop rotation, and lowest pyrolysis time (6 min). This corresponds to Run #3 with X1 at 6 min instead of 10 min, or Run #11 with non-stop rotation. Since the MW power sustains the resin decomposition reaction, it is logical that a higher power level will result in lower resin content after a set pyrolysis time. In the present case, a pyrolysis time of 6 min appears to have been sufficiently long to allow for the resin elimination percentage to reach a “maximum”. Furthermore, the nitrogen flow affects the rate of ejection of devolatilized material out of the reactor volume, which likely prevents the precipitation or condensation of carbon and hydrocarbon products inside the reactor. Finally, the rotation frequency appears to not have been a significant factor in promoting a complete resin devolatilization as the reactor size may have been sufficiently small to allow it to obtain a high temperature throughout the material and as the two other parameters have a more significant influence (power and flow rate). Figure 4 shows the histogram of the residual weight after pyrolysis for each run. Figure 5 shows the histogram of Y3, i.e., the resin elimination for each run, expressed as a percentage. 

### 3.3. Post-Pyrolysis Treatments

#### 3.3.1. Utilizing Quartz Sand Abrasion in MAP

**Figure 6:**
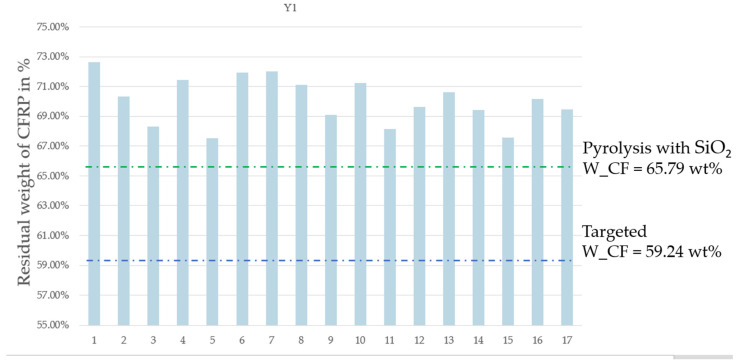
Residual weight of CFRP samples after MAP (Y1) in comparison with CFRP pyrolysis with SiO_2_.

Table 4 shows an average weight loss of CFRP (Y2) of 29.96% which means 10.80% of char and non-converted resin remain on the surface of the carbon fibers following MAP pyrolysis. The average residual carbon fiber weight after pyrolysis (Y1) is 70.04%. During experiments with quartz sand, two runs of CFRP MAP were performed under the pyrolysis conditions presented in Section 2.3. (a). The average residual carbon fiber weight after pyrolysis using quartz sand was 65.79%, which is shown in the green dotted line in Figure 6. The results show that the use of quartz sand during pyrolysis reduced the residual weight of CFRP after pyrolysis (Y1) from 10.80% to 6.55%. This change is significant as this is 40% of the remaining char and non-converted resin that could be removed using quartz sand during MAP pyrolysis. Therefore, the usage of quartz sand during pyrolysis enhanced the resin elimination percentage (Y3). The fiber content determined through chemical digestion (W_CF) is depicted as a blue dotted line in Figure 6 (Wf = 59.24%). 

#### 3.3.2. Chemical Digestion Using Sulfuric Acid and Hydrogen Peroxide Mixture

**Figure 7:**
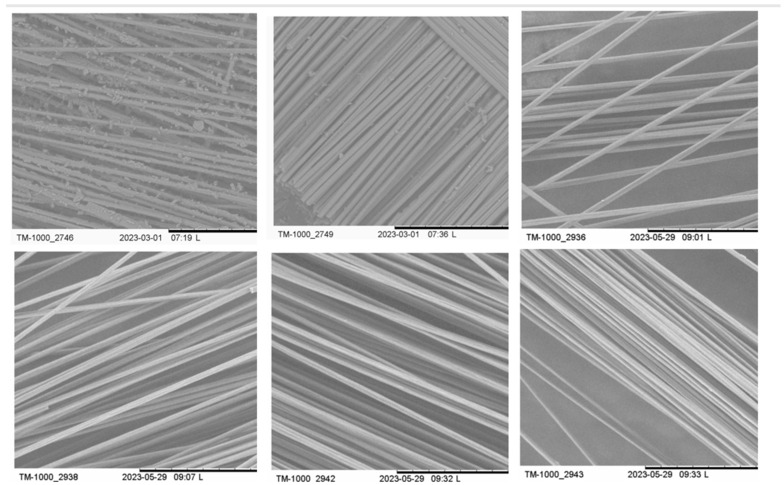
SEM of initial CFRP (#2746), after MAP (#2749), and after MAP and chemical digestion of the pyrolytic carbon residues after MAP (#2936, #2938, #2942, #2943).

After MAP, 10.80% of the weight fraction of the residue remained on the surface of the carbon fibers. These residues can be attributed to the char or unreacted resin. Thus, the sulfuric acid and hydrogen peroxide mixture was shown to efficiently digest both the carbon black and non-reacted residual resin. The digestion of char and non-reacted resin after MAP led to the recovering of clean carbon fibers, as shown in the SEM results in Figure 7. Furthermore, the XPS results presented in Section 3.4 confirm the absence of residue on the surface of the recovered carbon fibers after chemical digestion.

#### 3.3.3. Thermal Oxidation

- Thermal oxidation using microwave applicator oven

Following MAP, thermal oxidation was performed using a microwave applicator under an air atmosphere. Three durations were tested: 5, 10, and 15 min. Table 6 shows the sample descriptions and the average results obtained. The pyrolysis conditions were P1_3 (X1 = 1), which corresponds to a pyrolysis duration of 6 min, a nitrogen flow of 2.9 L/min, a power level of 100% (914 W), and non-stop rotation. The post-pyrolysis conditions were as follows: (1) the duration as described in Table 6; (2) air flow at 1.62 L/min; (3) power level at 100% (914 W); and (4) a variable rotation speed. Ten minutes provided the preferred behavior, as five minutes led to resin remaining on the surface of the recycled fibers and fifteen minutes led to evidence of oxidation when analyzing the residual weight after thermal oxidation. The MAP sample subjected to 10 min of thermal treatment using a microwave applicator resulted in recycled carbon fibers, exhibiting a texture comparable to that of virgin carbon fibers, as outlined in the XPS section that details the elemental constitution (Section 3.4). The results can be further refined by identifying the optimal duration between 10 and 15 min.

b. Thermal oxidation using a conventional oven

**Figure 8:**
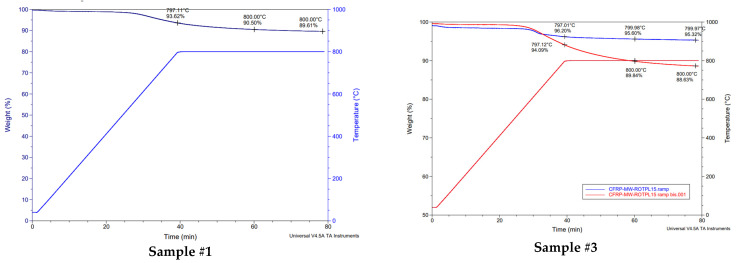

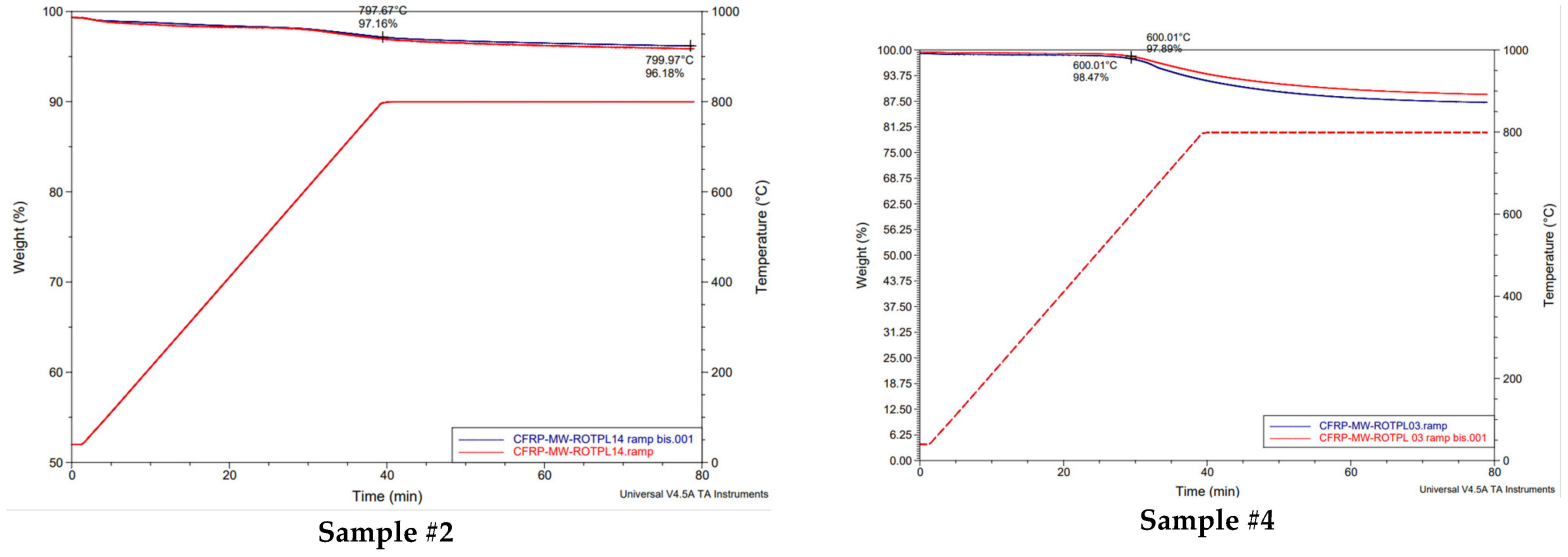
TGA curves of samples #1 to #4 after MAP.

**Figure 9:**
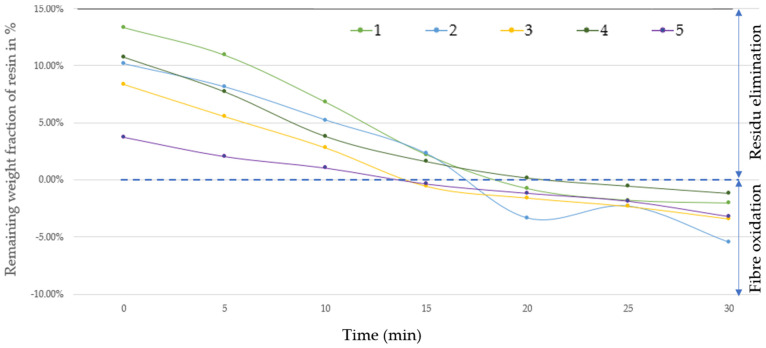
Thermal degradation curves of samples #1 to #5 during thermal oxidation treatment in a conventional furnace at 540 °C after MAP.

From the seventeen pyrolyzed samples, five samples were selected for post-pyrolysis treatment using thermal oxidation in a conventional furnace under an air atmosphere. A description of the selected samples is presented in Table 7. Pyrolyzed samples were first analyzed using TGA, as shown in Figure 8. Subsequently, the corresponding samples were exposed to a post-thermal treatment at 540 °C under an air atmosphere from 0 to 30 min. The corresponding results are presented in Figure 9. Therein, it is possible to observe that the optimal post-treatment duration using a conventional oven for the thermal treatment was around 18 min at 540 °C. A prolonged thermal treatment duration led to fiber oxidation. In fact, this result is consistent with previous studies, which demonstrate that the thermal decomposition of CFRP in an inert atmosphere primarily depends on the process temperature, while oxidation processes are influenced by both time and temperature [37].

### 3.4. XPS Results

X-ray photoelectron spectroscopy (XPS) was utilized to examine the chemical composition of the surfaces of the recycled carbon fibers. Table 8 shows the identification of samples used for XPS analysis. Detailed compositions and proportions of the elements are outlined in Table 9, While Table A1 in Appendix A shows the detailed composition of functional groups on the carbon fiber surfaces. The spectra obtained from both the virgin and recycled carbon fibers exhibited three prominent peaks, corresponding to carbon (C), oxygen (O), and nitrogen (N) at bonding energies of 284.6 eV, 532.9 eV, and 400.2 eV, respectively. These results correlate well with those in previous studies [38,39]. A comparison of the uncoated carbon fiber reference CFRP_D2584_1h_540C and the coated virgin carbon fiber revealed a slight difference in the C peak, with binding energies of 284.6 eV and 285 eV, respectively. In a similar study, the binding energy of 285 eV was associated with the coated virgin carbon fiber, while 284.8 eV was associated with graphite [39]. This enabled the differentiation of residuals on the surface of the carbon fibers, for example, the coating, carbon black, non-reacted resin, and uncoated carbon fiber [39]. This variance in binding energy, along with the quantification of the functional groups, is crucial for identifying the conditions that result in clean surfaces and polar groups containing oxygen. The N peak observed in the uncoated carbon fiber reference CFRP_D2584_1h_540C originates from the nitrogen atoms present in the carbon fiber itself, which are remnants of the polyacrylonitrile-based precursor, as well as from the amine (NH2) functional groups [40]. From the quantification results of the functional groups (Table 9), MAP samples exhibited carbon black remaining on the surface, with 28.7% being measured for the MAP sample and 21.3% for the MAP sample with quartz. This confirms that the utilization of quartz sand during MAP reduces the presence of carbon black on the fiber surfaces after microwave pyrolysis. 

In addition, it is noteworthy that 10 min of thermal oxidation using a microwave applicator is sufficient to achieve a clean fiber surface, the elemental composition of which closely correlates with the uncoated carbon fiber reference. The virgin carbon fiber and the uncoated carbon fiber reference CFRP_D2584_1h_540C exhibit a slightly higher O/C ratio (14.99%) in comparison to the recycled ones. The O/C ratio reflects the presence of oxygen-containing functional groups on the carbon fiber surfaces, indicating the extent of chemical bonding sites available for interaction with epoxy resin [40]. The higher O/C ratio in the carbon fiber references in comparison to the recycled carbon fibers suggests that treatment processes slightly change the carbon fiber surface morphology, by potentially breaking down oxygen-containing functional groups. 

Furthermore, the samples treated using chemical digestion exhibited an oxygen group distribution and O/C ratio similar to that of the coated and non-coated carbon fiber references. This suggests that the chemically digested samples had more active surfaces, providing more sites for robust chemical bonding with the polymer matrix. Finally, the lower oxygen content in the sample thermally treated for 10 min using a microwave applicator suggests the potential for optimization. Possible solutions may include increasing the oxygen flow rate or extending the post-thermal treatment duration to 12 min. In summary, the XPS analysis provided valuable insights into the chemical composition of the surfaces of recycled carbon fibers, elucidating the impact of various treatments on the surface properties and functionality.

## 4. Conclusions

This article presents an eco-friendly method for reclaiming carbon fiber-reinforced polymers (CFRPs). The study aimed to investigate the key parameters affecting the microwave-assisted decomposition of CFRPs. Through numerous experiments using microwave-assisted pyrolysis (MAP), factors such as the inert gas flow, power level, On/Off rotation frequency, and reaction duration were explored. The results show that the developed models accurately predicted the main influencing parameters in the microwave-assisted decomposition of CFRPs. The optimal operating conditions were found to be a nitrogen flow of 2.9 L/min and a power level of 914 Watts (100% power). Additionally, it was observed that the On/Off rotation frequency of the reactor vessel and increasing the reaction time beyond 6 min did not significantly affect the resin elimination percentage in comparison to the two other parameters, i.e., power and flow rate. The optimum conditions led to a maximum resin elimination percentage of 79.6%. After successful MAP, various post-pyrolysis treatments were used to remove char and non-reacted resin from the surface of the recovered carbon fibers, namely, thermal oxidative treatment using a microwave (MW) applicator, thermal oxidative treatment in a conventional furnace, mechanical abrasion with quartz sand, and chemical digestion. Among these techniques, thermal oxidation and chemical digestion were found to be the most efficient, leading to 100% of the carbon black content on the surface of recovered carbon fibers being eliminated. The efficiency of the post-treatments was established using SEM evaluations and the XPS analysis of the recovered carbon fibers. In conclusion, using a microwave applicator, the complete carbon fiber recovery process can be achieved in just 16 min, i.e., approximately 6 min for pyrolysis and 10 min for thermal oxidation. Given the environmental crisis and the goal of achieving carbon neutrality by 2050, this technology offers a less energy-intensive and environmentally friendly alternative to conventional pyrolysis technologies, as the heating mechanism involved induced less energy loss [27]. The products generated avoid the extraction of petroleum materials, thereby reducing greenhouse gas emissions. The production of recycled carbon fiber is significantly less expensive than the production of virgin fibers, costing USD 18 to 26 per kilogram compared to USD 35 to 65 per kilogram. Additionally, the process of producing recycled carbon fiber emits substantially less CO_2_, producing 4.65 tonnes of CO_2_ per tonne of recycled fiber versus 29.45 tonnes of CO_2_ per tonne of virgin fiber [7,8]. Limitations of such a study include the assumptions and fixed parameters used for the optimization study, as presented in detail in Section 2.2. Future work will consist in the refinement of the identified parameters that have a positive influence on the CFRP decomposition, i.e., flow rate and the level of power.

## Figures and Tables

**Table 1:** Fixed parameters and corresponding values.

Fixed Parameter Description	Numerical Value
Rotary evaporator speed of rotation	40 rpm
Sample mass	15 g
Feedstock composition	40.76% weight fraction of resin content (Wm_ini)
Feedstock size (square shape)	1.5 cm × 1.5 cm
Flask size	250 mL
Reaction flask angle in connection with the rotovap	15 degrees

**Table 2:** Variable parameters and corresponding values.

Variable and Unit		X1	X2	X3	X4
		Pyrolysis Time	N2 Flow Rate	MW Power	On/Off Frequency
		min	L/min	%	Number per Run
Level	1	6	0.5	50	0
	2	8	1.5	80	2
	3	10	2.9	100	4

**Table 3:** Resulting experimental plan using JMP 16 software.

Run	X1	X2	X3	X4
1	10	0.5	50	4
2	10	2.9	50	0
3	10	2.9	100	0
4	10	0.5	80	0
5	6	2.9	80	0
6	6	0.5	50	0
7	6	2.9	50	4
8	6	0.5	80	4
9	8	1.5	80	2
10	8	1.5	80	2
11	6	2.9	100	4
12	10	2.9	80	4
13	8	1.5	80	2
14	10	0.5	100	4
15	6	2.9	80	1
16	8	1.5	80	2
17	6	0.5	100	0

**Table 5:** Estimation of coefficients, t ratio and tabulated *p*-value using JMP software.

Source	Estimation	Standard Error	t Ratio	*p*-Value	Prob. > |t|
Flow_rate	3.363360	0.692071	4.86	0.00039	0.0004
Power	2.800000	0.875871	3.20	0.00768	0.0077
Rotation_Freq	−1.880026	0.692071	−2.72	0.01873	0.0187
Time	−1.080026	0.692071	−1.56	0.14460	0.1446

**Table 6:** Identification of samples used for thermal oxidation in microwave oven and the obtained results.

#ID	Nomenclature	Time (min)	Y1 %	Y2 %	Y3 %
1	P1_3(X1 = 1) − OXY 5	5	64.53	35.47	87.03
2	P1_3(X1 = 1) − OXY 10	10	62.96	37.04	90.87
3	P1_3(X1 = 1) − OXY 15	15	53.14	46.86	114.96
4	P1_3(X1 = 1) − OXY 15	15	55.90	44.10	108.19

**Table 7:** Identification of samples used for thermal oxidation in conventional oven.

	Nomenclature	Description
1	CFRP_MW__P1_1	MAP sample, Run #1 per Table 3
2	CFRP_MW_P1_14	MAP sample, Run #14 per Table 3
3	CFRP_MW_P1_15	MAP sample, Run #15 per Table 3
4	P1_3(X1 = 1)	1st repetition of optimum MAP corresponding to P1_3(X1 = 1)
5	CFRP_MW_Pyr_Oxy_10 min	P1_3(X1 = 1) followed by 10 min of oxidation in microwave oven

**Table 8:** Identification of XPS samples.

ID	Description
Virgin CF	Coated virgin carbon fiber reference, HTS40 Carbon fiber
CFRP_MW_PL01_15	MAP sample, RUN #15 per Table 3
CFRP_MW_quartz_sand	Quartz sand MAP sample as described in Section 2.3. (a)
CFRP_D3171B	CFRP digested following ASTM 3171 B as described in Section 2.3. (b)
CFRP_D2584_1h_540C	CFRP cleaned in a conventional oven, under air, at 540degC, for 1 h.
CFRP_MW_Pyr_Oxy_10 min	MAP sample, P1_3(X1 = 1) followed by 10 min of oxidation in microwave oven.

**Table 9:** Element content in recycled carbon fibers.

	Element				
	C(%)	O (%)	N(%)	Other * (%)	O/C(%)
Virgin CF	85.40	12.80	1.30	0.5	14.99
CFRP_MW_rot_PL01_15	88.80	4.20	6.40	0.6	4.73
CFRP_MW_quartz_sand	90.00	4.40	4.50	1.1	4.89
CFRP_ASTMD3171B	83.60	12.50	3.40	0.5	14.95
CFRP_D2584_540C-1h	77.40	11.60	10.00	1.0	14.99
CFRP_MW_Pyr_Oxy10min	81.70	6.50	10.80	1.0	7.96

*: This total fraction represents traces of three additional elements which are not relevant to the current study: silicon, sulfur, and boron.

## Data Availability

Data are contained within the article.

## Appendix A

**Table A1:** Functional groups in recycled carbon fibers.

Element	C1s							N1s				O1s				
Functional Group	C=C (sp2)	C-C (sp3)	C-N	C-O, C-O-C	C=O	O-C=O	C=C (sp2) ^(1)^	N ^(2)^	C-N	N ^(3)^	(sp2) ^(4)^	O ^(5)^	C=O, O-C=O	C-O, C-O-C	O-C=O	O ^(6)^
	(%)	(%)	(%)	(%)	(%)	(%)	(%)	(%)	(%)	(%)	(%)	(%)	(%)	(%)	(%)	(%)
Binding energy (Ev)	284.6	285.0	285.7	286.5	287.9	289.2	291.2–294.0	398.8	400.2	400.9	402.8	530.7	531.5	532.9	533.7	536.4
Virgin CF		63.0		21.3		0.8			1.4					13.5		
CFRP_MW_rot_PL01_15	42.6	28.7	12.4	1.9		0.9	2.8	2.4		3.6			0.6	3.1	0.9	
CFRP_MW_quartz_sand	56.6	21.3	7.4	1.6		0.8	2.7	1.8	2.6				1.6	3.0	0.6	
CFRP_ASTMD3171B	53.7	14.6	6.6	3.6	1.3	2.4	1.4	0.7		2.5			6.1	3.5	3.6	
CFRP_D2584_540C-1h	64.3		6.1	3.8	1.9	0.9	1.0	4.8		3.9	0.9		3.8	5.4	2.0	1.5
CFRP_MW_Pyr_Oxy10 min	67.1		8.5	1.3	1.7	1.3	3.0	4.6		4.3	1.1	1.9	1.1	1.4	1.5	1.1

^(1)^ Shake up (π→π*) of C=C (sp2), ^(2)^ Pyridinic N, ^(3)^ Pyrrolic N, ^(4)^ Sp2 N or shake up, ^(5)^ Quinone, ^(6)^ Shake up.
